# A rotamer relay information system in the epidermal growth factor receptor–drug complexes reveals clues to new paradigm in protein conformational change

**DOI:** 10.1016/j.csbj.2021.09.026

**Published:** 2021-09-27

**Authors:** Tareq Hameduh, Michal Mokry, Andrew D. Miller, Vojtech Adam, Zbynek Heger, Yazan Haddad

**Affiliations:** a Department of Chemistry and Biochemistry, Mendel University in Brno, Zemedelska 1, CZ-613 00 Brno, Czech Republic; b Central European Institute of Technology, Brno University of Technology, Purkynova 656/123, 612 00 Brno, Czech Republic; c Veterinary Research Institute, Hudcova 70, CZ-62100 Brno, Czech Republic; d KP Therapeutics (Europe) s.r.o., Purkyňova 649/127, Brno CZ-61200, Czech Republic

**Keywords:** EGFR, NSCLC, Tumour resistance, Tyrosine kinase inhibitor, Rotamer, Protein structure, Protein folding

## Abstract

Cancer cells can escape the effects of chemotherapy through mutations and upregulation of a tyrosine kinase protein called the epidermal growth factor receptor (EGFR). In the past two decades, four generations of tyrosine kinase inhibitors targeting EGFR have been developed. Using comparative structure analysis of 116 EGFR-drug complex crystal structures, cluster analysis produces two clans of 73 and 43 structures, respectively. The first clan of 73 structures is larger and is comprised mostly of the C-helix-IN conformation while the second clan of 43 structures correlates with the C-helix-OUT conformation. A deep rotamer analysis identifies 43 residues (18%) of the total of 237 residues spanning the kinase structures under investigation with significant rotamer variations between the C-helix-IN and C-helix-OUT clans. The locations of these rotamer variations take on the appearance of side chain conformational relays extending out from points of EGFR mutation to different regions of the EGFR kinase. Accordingly, we propose that key EGFR mutations act singly or together to induce drug resistant conformational changes in EGFR that are communicated *via* these side chain conformational relays. Accordingly, these side chain conformational relays appear to play a significant role in the development of tumour resistance. This phenomenon also suggests a new paradigm in protein conformational change that is mediated by supportive relays of rotamers on the protein surface, rather than through conventional backbone movements.

## Highlights

• EGFR kinase C-helix moves relate to side chain rotamers of 43 residues.
• Rotamer changes formed several clusters as part of two major information relays.
• Relay-IN identifies rotamers in active C-helix-IN/DFGin/BLAminus conformations.
• Triple mutants were in Relay-OUT with inactive C-helix-OUT/DFGout/BBAminus.
• Global rotamer moves can impact kinase activation and tumour resistance.

## Graphical abstract

**Figure:**
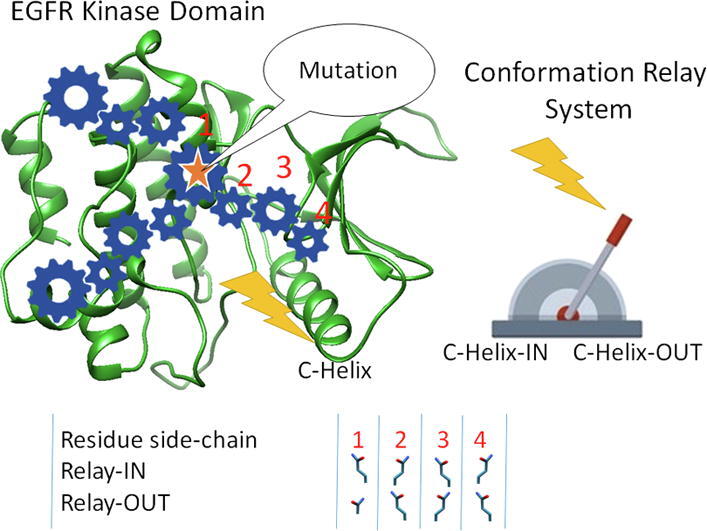

## 1 Introduction

Epidermal growth factor receptor (EGFR) protein was discovered in the late 1970s, and to this day it remains a primary target for anticancer therapy [1], [2]. Mutations and upregulation of EGFR are known mechanisms of both oncogenesis and therapeutic resistance. Active EGFR mutants escape the effects of chemotherapy via continuous proliferation and evasion of apoptosis, especially in the case of non-small cell carcinoma of the lung (NSCLC) [3]. Furthermore, EGFR is prevalent in other solid tumours including: breast, colon, renal, ovarian, and head-and-neck cancers [4]. EGFR is a member of the receptor tyrosine kinases (RTKs) cell surface receptor family [5], [6], and is also a member of the ErbB family, a four member family responsible mainly for regulating cell proliferation [6]. The primary function of EGFR is to mediate signals for differentiation, motility, and apoptosis [6]. Therefore, any irregular EGFR behaviour can easily become pro-oncogenic [7]. Pro-oncogenic mutations in EGFRs are widespread and linked to tumour overgrowth and resistance to chemotherapy [8].

EGFR comprises three protein domains, 1) an extracellular ligand-binding domain of 621 amino acid residues, 2) a transmembrane domain of 23 amino acid residues, and 3) a cytoplasmic domain comprising 542 amino acids [9], [10], [11]. The extracellular domain contains the ligand-binding pocket that mediates the open-close status of the receptor to signal transduction. The transmembrane domain is a single α helix. Finally, the cytoplasmic domain contains a juxtamembrane cytoplasmic subdomain, a tyrosine kinase subdomain and a C-terminal subdomain [12]. EGFR ligands include epidermal growth factor (EGF), amphiregulin (AREG), transforming growth factor (TGF) and epigen [13]. Other ligands are heparin-binding EGF (HB-EGF), epiregulin (EPR) and betacellulin (BTC) [14]. Ligand binding results in EGFR activation by either homodimerization or heterodimerization (EGFR-ErbB_2/3/4_) that leads to the intracellular modulation of different signalling pathways (MAPK, PI3K/Akt/mTOR or JAK/STAT) [15], [16]. In cancer, EGFR activation leads to the downstream initiation of different key cellular events involved in cellular growth, proliferation, invasion, metastasis and angiogenesis [17]. Overproduction of EGFR ligands can also drive cancer progression, however, that can be influenced by the cancer microenvironment [10], [15], [18], hypersensitization of tyrosine kinase subdomain [19], and EGFR overexpression [20]. In the rest of this article, the word “ligand” will be exclusively used to describe the compounds or drugs binding to the intracellular kinase domain, unless mentioned otherwise.

Chemotherapeutic approaches have focused on several aspects of the EGFR structure and mechanism of action. For example, monoclonal antibodies, such as cetuximab, have been devised to target the extracellular domain, as reported in treatments for bowel or head and neck cancer [21]. Inhibitors of the intracellular tyrosine kinase subdomain act to attenuate tyrosine kinase activated signalling pathways, and thereby trigger cancer cell death [22], with reports of success in NSCLC patients in terms of treatment and quality of life [23]. Unfortunately, it has become clear now that both monoclonal antibody-based agents and tyrosine kinase inhibitors (TKIs) are struggling to keep pace with the emerging of new EGFR mutations. While monoclonal antibodies are effective against wild type EGFR [24], they are much less effective against EGFR mutants (*i.e.*, exon 19 deletions and L858R mutation) detectable in 10–15% of Caucasian NSCLC patients and ∼ 50% of Asian patients [25], [26]. The first generation TKIs such as erlotinib and gefitinib were originally thought to be more robust against EGFR mutations. However, the emergence of the T790M mutation, in the EGFR ATP-binding site of the tyrosine kinase subdomain, was sufficient to curtail the efficacies of reversible first generation TKIs [27]. The T790M mutation is present in about 50%–60% of patients that develop chemoresistance to TKIs [28]. First generation TKIs were quickly replaced by irreversible second generation EGFR TKIs (pan-HER inhibitors), such as afatinib, dacomitinib and neratinib, that functionally inhibit wildtype and T790M EGFR mutants [29]. Thereafter, third generation EGFR TKIs were developed, such as osimertinib and rociletinib, to overcome this particular chemoresistance, with more efficacy and less side effects than observed with first and second generation inhibitors particularly owing to covalent binding to the C797 residue [26]. Unfortunately, the appearance of a C797S mutation in the tumours of patients treated with third generation TKIs rapidly curtailed the efficacy of these drugs [30], [31]. Accordingly, state-of-the-art fourth generation allosteric EGFR inhibitors, like EAI045 and EAI001, were created to target a different binding site in the EGFR kinase. These drug entities avoid the problems of both T790M and C797S mutations. However, the evidence suggests that such fourth generation inhibitors are insufficiently effective alone, for example EAI045 is only properly effective in combination with cetuximab [32], [33].

Clearly, resistance to chemotherapy is not just simply a function of EGFR mutations alone. EGFR-independent factors include the overexpression of additional growth factor receptors such as HER2, MET and FGFR, reduced NF1 expression, and overactivity of PI3K or B-Raf [28], [34], [35], [36]. Having said this, EGFR mutations are dominant, and thus EGFR remains a primary upstream target for cancer chemotherapy.

Recently exploited concepts in cancer like the game theory and the biological informational theory could help to understand this problem in more sophisticated way. The game theory in cancer, describes how cancer can take advantage of the dynamics of the tumour microenvironment for its own survival by different ways, for instance, tumour therapy resistance [37]. On the other hand, the biological information theory in cancer, could help us to understand how cancer cells can maintain their signal transduction specificity and the amount of information transmitted with different mutations. Further, it explains how cancer develops resistance to hold the integrity of its informational system, which will be of interest of cancer cells survival [38]. Accordingly, there is a major unmet need to understand the molecular mechanisms of EGFR mutation-induced resistance to chemotherapy. In the light of increasing numbers in deposited EGFR kinase 3D structures, it has become a challenge in structure-based drug design to make a choice without fully understanding the variations involved at the molecular level. In a previous study, we have identified C-helix in the N-lobe of EGFR kinase domain as the major structural variation occurring in EGFR kinase complexes with ligands based on analysis of backbone movements [39]. The objective of this work is to investigate the relationship between EGFR kinase domain rotamer variations, mutations, C-helix movement, and kinase activation (DFG domain movements). By taking advantage of our recently developed code for rotamer analysis [40], here, we attempted to shed the light on the biological relevance of global rotameric changes in the EGFR kinase.

## 2 Material and methods

### 2.1 Dataset processing

Using the keyword “EGFR”, RCSB protein databank (www.rcsb.org) database search resulted in 260 structures. All entries that did not cover the kinase domain of EGFR were excluded. Entries without inhibitors were also excluded except for four wildtype entries: 1M14, 2GS2, 3GOP, 4TKS. Only chain A was retrieved (the number of chains ignored were 28 from all structures). In total, 116 EGFR kinase 3D structures spanning 714–950 (Uniprot ID P00533-1) were trimmed and further studied. The two lobes of the kinase domain were identified as N-lobe (spanning 714–795) and C-lobe (spanning 796–950), and the ligands were salvaged.

The 83 ligands salvaged in these structures were previously classified by us according to the primary and secondary heterocyclic group into the following classes: 1 Antibiotic, 2 Benzimidazoles, 6 Furopyrimidines, 2 Indolocarbazoles, 7 Purines, 1 Pyrazine, 7 Pyrazolopyrimidines, 3 Pyridones, 34 Pyrimidines, 1 Pyrimidopyridone, 8 Pyrrolopyrimidines, 7 Quinazolines, 1 Quinolines, and 2 Thiazoles.

### 2.2 Visualization

Visualization of Protein and ligand 3D Structures was done in UCSF Chimera (version 1.10.2). The matchmaker plugin was used for superposition of all heavy atoms *via* BLOSUM-62 scoring matrix and Needleman-Wunsch alignment algorithm. Structure rendering and animation were done in UCSF Chimera using the command line. Graphics were processed using MS Powerpoint and Adobe Photoshop.

### 2.3 Structure fitting and cluster analysis

Structure fitting and cluster analysis were done in R language (Version 3.6.1, The R foundation for Statistical Computing, Austria) using rmsd() function from Bio3D library (Grant lab, University of California, San Diego, USA) and agnes() function from Cluster library (Martin Machler, ETH Zurich, Switzerland) using the Ward method for root mean square deviations (RMSD) dissimilar matrix, respectively. The Ward method of clustering, which is also known as the minimum variance method, is general purpose method of clustering that starts with *n* clusters (each containing a single structure), then these clusters are combined in each step (minimizing the variance) until all structures are contained within a single cluster. RStudio Version 1.2.5001 (RStudio, Inc.) was used for coding and obtaining results.

### 2.4 C-helix and DFG domain analysis

C-helix movement and DFG domain clustering were measured by two techniques. 1) the angles of the helix axis (residues 756–767) between each structure and a reference structure (Reference PDB IDs: 3gop and 1m14) were measured using a command line in UCSF Chimera after superposition of 3D structures. 2) Active and inactive kinase structures based on clusters of DFG domain torsions were estimated according to the new nomenclature of Modi and Dunbrack [41]. Briefly, the DFG cluster is comprised of the Ramachandran regions (A, alpha; B, beta; L, left) of the Tyr854, Asp855 and Phe856 in addition to the first side chain angle of Phe856 (minus, plus, trans). The most common active DFGin cluster is BLAminus (beta Tyr854, left Asp855, alpha Phe856 and minus Phe856 side chain), whereas the most common inactive DFGout cluster is BBAminus (beta Tyr854, beta Asp855, alpha Phe856 and minus Phe856 side chain).

### 2.5 Comparative rotamer analysis

Comparative rotamer analysis was done in R language according to our previously published method [40]. Briefly, using Bio3D library, structures were loaded via read.pdb() function and torsional angles were calculated via torsion.pdb() function. Classification of rotamers was done according to the Richardson’s Penultimate rotamer library [42], using IF/ELSE statements as previously described [40]. Rotamer nomenclature is based on the side chain torsional angles (χ1 torsion between N, Cα, Cβ and Cγ atoms, χ2 angle between Cα, Cβ, Cγ and Cδ atoms, etc.). The angle modes were used to classify the three main classes as plus/trans/minus (*i.e.*, *p*, *t*, *m* for +60°, 180°, and -60°, respectively). For atoms in side chains other than carbon, the angles modes were shifted and were replaced with explicit angle mode (*e.g.*, m-80 rotamer for Asn described χ1 = -60° and χ2 = -80° modes). In this manuscript, the degree symbol for explicit angles was conveniently removed from nomenclature for coding purposes. The R scripts, which are still not optimized as a package, are available for academic purposes upon request from the corresponding author.

Chi square test of independence was used to construct crosstabs between groups of structures and residue rotamers and to determine if there was any association between the variables in IBM SPSS Statistics 21 program (IBM Corporation, Armonk, New York, USA). A *p*-value below 0.05 was considered significant.

## 3 Results and discussion

### 3.1 Rotameric differences between EGFR kinase structures

In the past two decades, an impressive wide range of TKIs targeting EGFR have been devised [39]. At the same time, a large number of EGFR-ligand complexes have been deposited in the database every year, although these structures are not necessarily representative of the latest trends in EGFR inhibitor design due to the time required for X-ray crystallography experiments. Nevertheless, the availability of hundreds of similar such structures does provide statistical confidence in the validity of comparative structure analysis regarding conformational changes.

Accordingly, we obtained and trimmed 116 EGFR structures for fitting and clustering analysis using RMSD for all atoms.

**Fig. 1:**
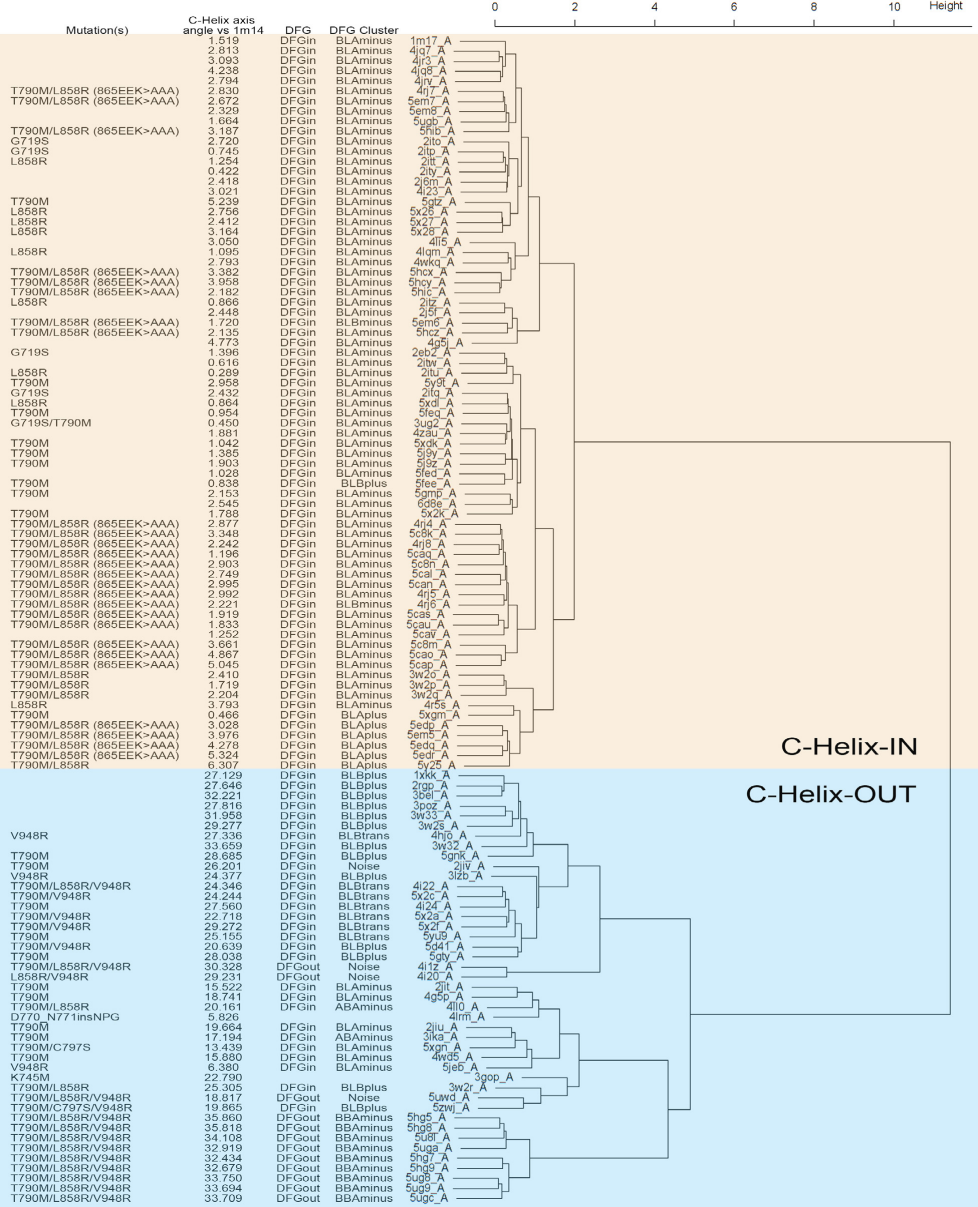
Cluster analysis of RMSD between EGFR kinase structures spanning 714–950 residues (numeration according to Uniprot ID P00533-1). The C-helix-IN conformation clan of structures is shown on top in tanned colour whereas the C-helix-OUT conformation clan of structures is shown below in light blue colour. C-helix-IN clan showed mostly the active C-helix-IN (DFGin/BLAminus) conformations. The triple mutants in the C-helix-OUT clan showed mostly the inactive C-helix-OUT (DFGout/BBAminus) conformations. C-helix orientation was estimated by the helix axis angle against the helix axis of a reference C-helix-IN (PDB ID 1m14). (For interpretation of the references to color in this figure legend, the reader is referred to the web version of this article.)

Cluster analysis showed two main clans of structures (Fig. 1) similar to our previous analysis of the N-lobe of EGFR kinase [39]. The first clan of 73 structures was larger and comprised mostly of the C-helix-IN conformation and two or less mutations. While the second clan of 43 structures was correlated with the C-helix-OUT conformation and three or less mutations. In our previous work, the focus was on RMSD values calculated for Cα-backbone movements, here we chose to study both backbone and side chain movements. Initially, in order to quantify the C-helix axis movement, two reference structures were used to define C-helix-IN (PDB ID 1m14) and C-helix-OUT (PDB ID 3gop) conformations. Interestingly, the C-helix axis angles for 1m14, and other C-helix-IN conformations, were in the range > 0° to 6°, while C-helix angles for 3gop, and other C-helix-OUT conformations, were in the range of 6° to 33° degrees (average ∼ 25° degrees) (Supplementary Table 1), although in different outward directions.

**Fig. 2:**
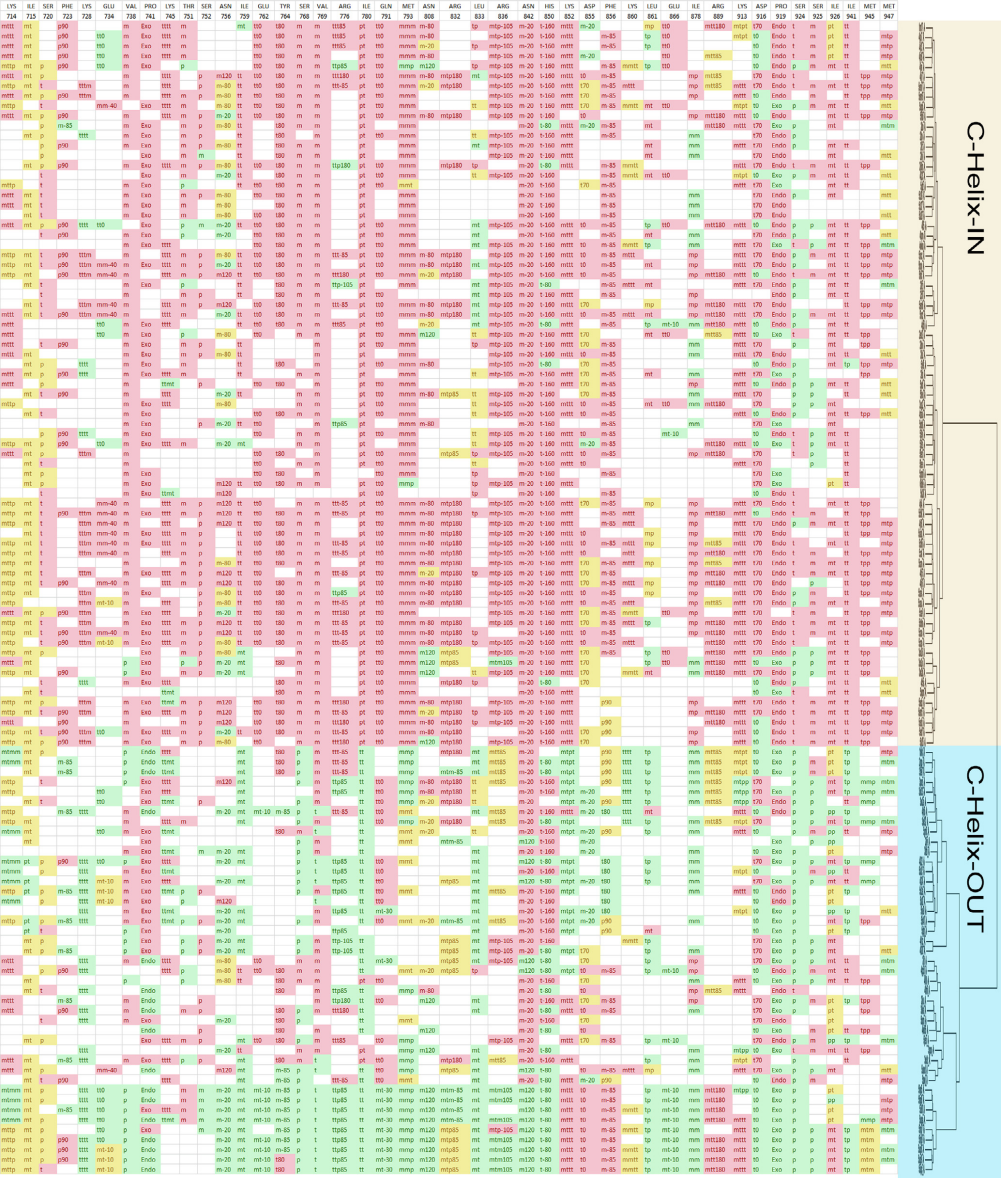
Differential Color map of rotamers in residues of C-helix-IN and C-helix-OUT clans. Rows represent 116 EGFR structures while columns represent 43 residues of the relay information system. Skewed cluster tree is shown for convenience.

For more insight, we then performed a deep rotamer analysis (Supplementary Table 1). Nearly 43 residues (18%) of the total of 237 residues spanning the studied kinase structures showed significant rotamer variations between the C-helix-IN and C-helix-OUT clans (Table 1 and Fig. 2). In particular, six residues spanning the C-helix (namely, Asn756, Ile759, Glu762, Tyr764, Ser768, and Val769) exhibited significant rotamer variations between C-helix-IN and C-helix-OUT conformations. In addition, two residues of the DFG domain (Asp855 and Phe856) also displayed significant rotamer variations between the C-helix-IN and C-helix-OUT clans.

**Table 1:** Top frequent differential rotamers between C-helix-IN and C-helix-OUT clans (count is shown after each rotamer).

Residue		C-helix-IN clan			C-helix-OUT clan			IN	OUT	Total	p-value
		1	2	3	1	2	3				
Lys	714	mttp 28	mttt 21	ttmt 2	mttp 11	mtmm 11	mttt 6	52	32	84	<0.001
Ile	715	mt 52	mm 11	mp 1	mt 24	pt 5	mm 5	64	36	100	0.012
Ser	720	t 35	p 22	m 9	p 18	m 11	t 7	66	36	102	0.003
Phe	723	p90 30	m-85 1	m-30 1	m-85 9	p90 7	m-30 3	32	20	52	<0.001
Lys	728	tttm 24	ttpt 7	tttt 6	tttt 23	ttpt 4	pttt 3	41	33	74	<0.001
Glu	734	mm-40 13	tt0 9	mt-10 2	tt0 10	mt-10 6	pt-20 3	27	21	48	0.016
Val	738	m 65	t 4	p 2	p 18	m 15	t 7	71	40	111	<0.001
Pro	741	exo 52			exo 21	endo 17		52	38	90	<0.001
Lys	745	tttt 37	ttmt 4	ttpt 2	tttt 12	ttmt 10	ttpt 5	43	30	73	0.001
Thr	751	m 45	p 7	t 2	p 8	m 7	t 5	54	20	74	<0.001
Ser	752	p 41	t 16	m 2	p 10	t 8	m 6	59	24	83	0.005
Asn	756	m-80 19	m120 15	m-20 13	m-20 20	m-80 3	m120 3	49	27	76	0.001
Ile	759	tt 43	tp 6	mt 5	mt 28	mm 5	tp 3	59	41	100	<0.001
Glu	762	tt0 52			mt-10 9	tp10 5	tt0 3	52	17	69	<0.001
Tyr	764	t80 57			t80 14	m-85 10	m-30 3	57	27	84	<0.001
Ser	768	m 64			p 30	m 6	p 2	64	38	102	<0.001
Val	769	m 72			m23	t 17	p 1	72	41	113	<0.001
Arg	776	ttt-85 14	ttt180 6	ttt85 4	ttp85 18	ttt-85 5	ptt85 2	34	31	65	0.001
Ile	780	pt 72	mm 1		tt 38	pt 3	mt 1	73	42	115	<0.001
Gln	791	tt0 62			tt0 17	mt-30 11		62	28	90	<0.001
Met	793	mmm 67	mmp 2	mmt 1	mmp 21	mmt 9	mtm 1	70	31	101	<0.001
Asn	808	m-80 28	m-20 6	m120 6	m120 12	m-20 5	t30 5	41	26	67	<0.001
Arg	832	mtp180 29	mtt180 6	mtp85 4	mtp85 11	mtm-85 7	mtp180 6	41	28	69	<0.001
Leu	833	tp 16	tt 12	mt 11	mt 31	tt 4	pp 1	39	37	76	<0.001
Arg	836	mtp-105 56	mtm105 1	mtm180 1	mtt85 9	mtm105 7	mtp-105 5	58	28	86	<0.001
Asn	842	m-20 73			m-20 28	m120 15		73	43	116	<0.001
His	850	t-160 67	t-80 6		t-80 23	t-160 17	m80 3	73	43	116	<0.001
Lys	852	mttt 60			mtpt 17	mttt 15	mtpp 1	60	33	93	<0.001
Asp	855	t70 23	t0 21	m-20 4	t0 14	m-20 10	t70 6	48	30	78	0.006
Phe	856	m-85 53	p90 3		m-85 14	p90 9	t80 7	56	30	86	<0.001
Lys	860	mttt 13	mmtm 9	mmtt 7	tttt 8	mmtm 6	mmtt 6	36	24	60	<0.001
Leu	861	mt 13	tp 9	mp 8	tp 26	mt 2	mp 1	36	30	66	<0.001
Glu	866	tt0 13	mt-10 2	tm-20 1	mt-10 11	mm-40 1	pt-20 1	17	13	30	<0.001
Ile	878	mp 30	mm 17	mt 10	mm 29	mp 4	mt 4	64	37	101	<0.001
Arg	889	mtt180 23	mtt85 7	mtt-85 2	mtt180 8	mtt85 8	mmt-85 4	33	25	58	0.043
Lys	913	mttt 48	mtpt 4	mttp 3	mttt 12	mtpt 7	mtpp 5	55	30	85	<0.001
Asp	916	t70 47	t0 25		t0 26	t70 15		72	41	113	0.003
Pro	919	endo 54	exo 13		exo 29	endo 9		67	38	105	<0.001
Ser	924	t 30	p 29	m 1	p 38	t 2		60	40	100	<0.001
Ser	925	m 28	t 19	p 13	p 18	t 11	m 10	60	39	99	0.025
Ile	926	mt 50	pt 5	mm 3	mt 18	pt 14	pp 7	59	39	98	<0.001
Ile	941	tt 61	tp 1		tp 19	tt 10	mt 5	62	35	97	<0.001
Met	945	tpp 37	tpt 4	ttm 2	mmp 7	tpp 6	mtm 5	44	29	73	<0.001
Met	947	mtp 28	mtt 13	mtm 3	mtm 10	mtp 7	mtt 3	44	20	64	<0.001

**Fig. 3:**
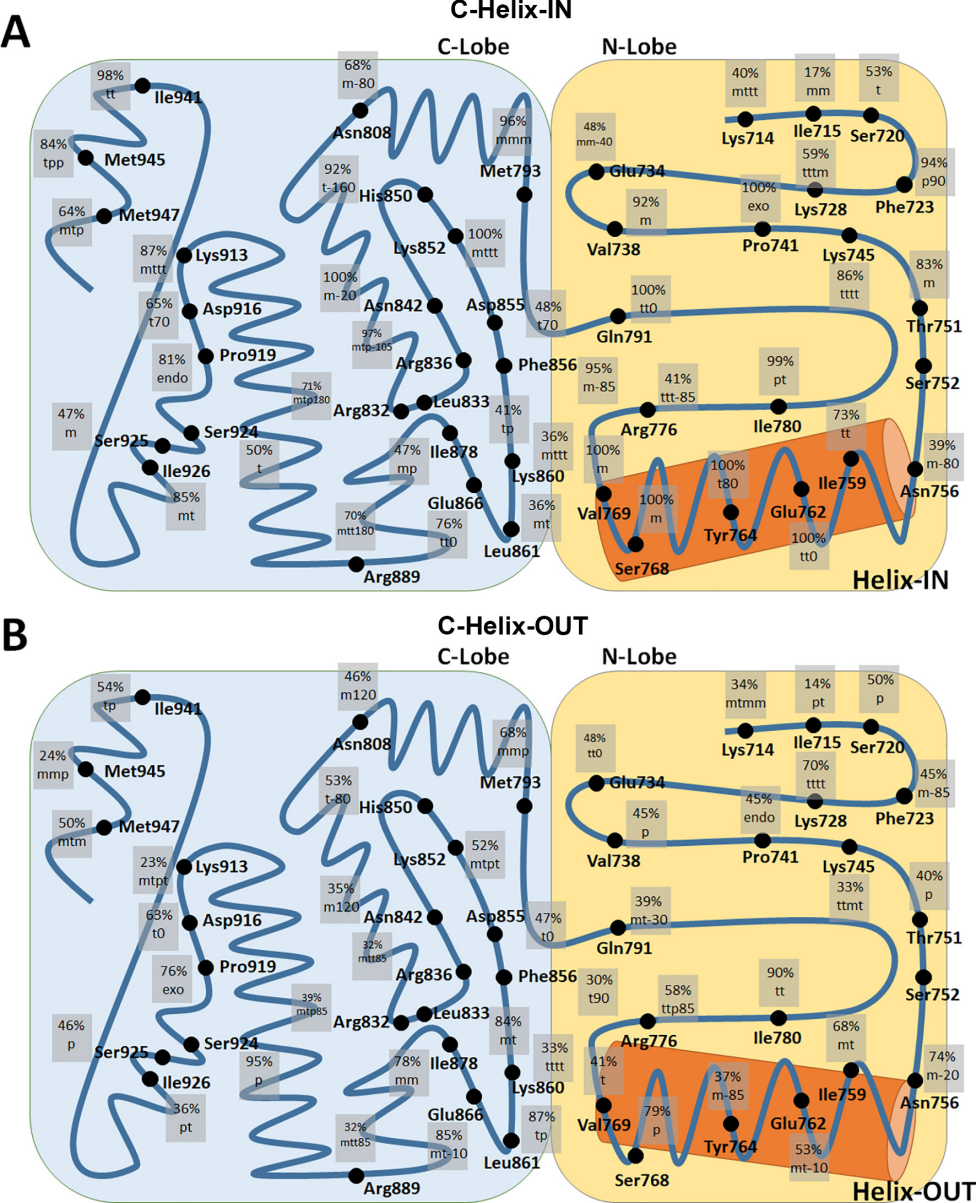
Schematic drawing of the EGFR kinase domain showing the frequencies of the top differentiating rotamers between C-helix-IN (A) and C-helix-OUT (B) clans. In some cases, the low percentage is actually representing the second top rank rotamer as statistically differentiating where the first top rank rotamer was similar (*e.g.*, in Ile715 the first top rank rotamer was mt in both relays).

**Fig. 4:**
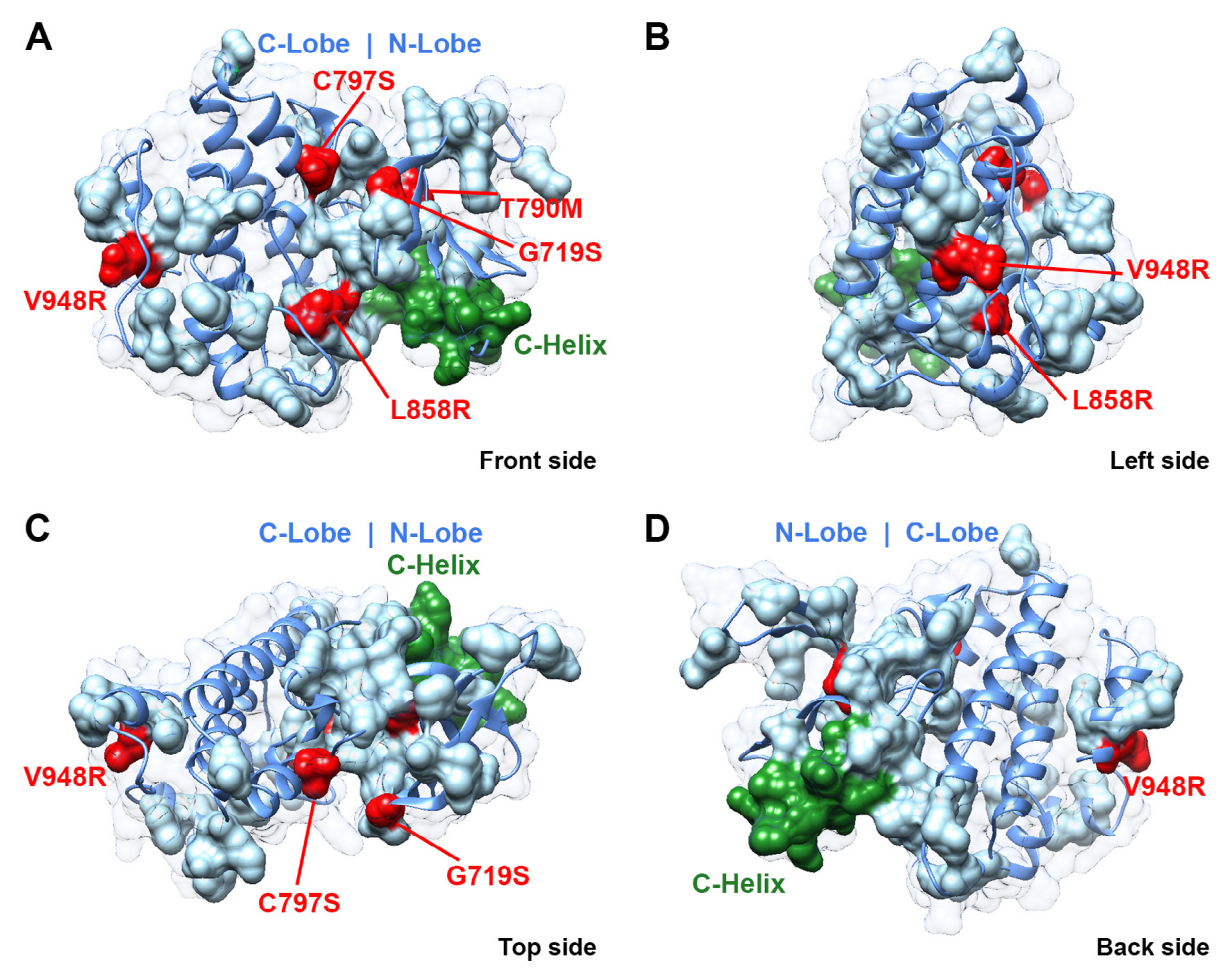
(A-D) EGFR kinase domain structure (PDB ID 5ugc) showing the information relay residues as solid light blue surface (C-helix in green and mutations in red). The rest of the kinase is shown in blue ribbon and transparent solvent accessible surface. Most of the relay is connected and fits the flow of information from mutation to the rest of the relay (note that V948R mutation connects to only few residues in the C-lobe, in which case the information from V948R is transferred by other means than rotameric moves such as backbone, water and allosteric effects). (For interpretation of the references to color in this figure legend, the reader is referred to the web version of this article.)

In general, three types of rotamer variations were observed between the C-helix-IN and C-helix-OUT clans: Firstly, variations comprising unique rotamers. Secondly, where one rotamer is common in both clans but the second ranked rotamer is different. Thirdly, where one rotamer is common in ∼ 100% of cases in one clan, while other rotamers dominate in the other clan. Rotamer differentiations were most clear with triple mutants (last ten rows at the bottom of Fig. 1, Fig. 2). Although, triple mutants were seen to share similar rotamers in their DFG domains, particularly at Lys852 and Phe856, and at other more distant residues (Arg889 and Lys913). Most importantly, the locations of these rotamer variations take on the appearance of side chain conformational relays extending out from points of mutation to different regions of the EGFR kinase (Fig. 3, Fig. 4**,** Supplementary Movie 1–3).

This poses the obvious question which is, can EGFR mutations act singly or together to induce drug resistant conformational changes in EGFR that are communicated *via* these side chain conformational relays? Furthermore, can rotamer variations on the protein surface also play role in allosteric mechanisms? In fact, given that most of the side chains in the 43 residues mentioned above are actually facing the protein surface (with access to water solvent), then any potential “flow of information” from rotamer to rotamer in such a dynamic environment must also involve torsional angle movements in both backbone and side chains in addition to surface network H-bonds or salt bridges with water molecules. Indeed, nearly 20 residues (∼47%) in the relay are located in the smaller N-lobe and thus are more exposed to water solvent. Furthermore, rotamer variations are observed across most types of amino acids (except for Cys and Trp, perhaps due to their general infrequency in proteins), and not only long chains amino acids (*e.g.* such as Arg and Lys) that may display alternative rotamers on the surface due to their flexibility alone [43], [44].

### 3.2 Resistance, mutations and EGFR relay systems

Tumours are known to develop two types of drug resistance, *i.e.*, the innate and the acquired. Innate resistance is defined as the failure of initial therapy due to various tumour mechanisms. Acquired resistance is defined as progression (*e.g.*, due to mutations) of the disease after a period of “clinical benefit” [45]. Acquired EGFR-TKIs resistance mechanisms vary according to TKI types, mutation types, and other factors. Before we highlight those EGFR mutations involved in acquired resistance mechanisms, it is important to emphasize on the nature of wildtype EGFR kinase in free and drug-bound forms. The catalytic activity of EGFR is regulated by three mechanisms: phosphorylation, autoinhibition, and allosteric binding [46]. Wildtype EGFR kinase is intrinsically autoinhibited in a similar way to the Src and CDK proteins, and interestingly, even though EGF ligand-free wildtype EGFR is not phosphorylated (not activated) this protein adopts the active form conformation in crystals. Indeed, unlike other tyrosine kinases, EGFR kinase is not as “tightly” autoinhibited, and can maintain an active conformation and basal activity at high concentrations of EGFR or ErbB2 heterodimer [47], [15]. In our previous study [39], we mentioned three wildtype PDB structures of ligand-free EGFR, namely, PDB IDs 1m14, 2gs2, and 4tks all showing the C-helix-IN (DFGin/BLAminus) conformation. Hence why 1m14 was selected as reference structure for the C-helix-IN clan. The C-helix-OUT conformation is found in 3gop which was used here as reference structure for the C-helix-OUT clan (even thought this does in fact comprise a K745M mutation).

James & Verkhivker, [46] described the following possible states of activation for wildtype EGFR (following examples with compounds complexed): (1) an inactive state (C-helix-IN/DFGout). (2) a Cdk/Src inactive conformation 1 (C-helix-OUT/DFGin), *e.g.*, PDB ID 1xkk. (3) a Cdk/Src inactive conformation 2 (C-helix-OUT/DFGout), *e.g.*, PDB ID 2rf9. And (4) an active state (C-helix- IN/DFGin), *e.g.*, PDB ID 2itx. In our C-helix-IN clan, 19 complexed structures were identified with wildtype EGFR in the active C-helix-IN (DFGin/BLAminus) conformation **(**Supplementary Table 1**)**. In the C-helix-OUT clan, nearly 7 complexed wildtype EGFR structures were found in the inactive C-helix-OUT (DFGin/BLBplus) conformation **(**Supplementary Table 1**)**.

The first and most common type of mutation in EGFR, the T790M mutation, occurs at exon 20 of the *EGFR* gene which is responsible for > 60% of the acquired resistance cases in NSCLC. Here, we observed T790M single mutants with the DFGin conformation in both C-helix-IN (∼10 structures) and C-helix-OUT (∼10 structures) clans, with nearly 12 BLAminus conformations **(**Supplementary Table 1**)**. Hence, we would suggest that the T790M mutation is not critical in triggering the relay of a “flow of information” from rotamer to rotamer to desensitize EGFR kinase to TKIs. Such a statement is not surprising given that T790M mutation involves just the replacement of threonine – a local gatekeeper residue and important determinant of inhibitor specificity in the ATP-binding pocket – to methionine. This residue replacement decreases first and second generation TKI binding affinities for the ATP-binding pocket, owing to increased steric hindrance, and increases the ATP-binding affinity; thus, enhancing competition for binding between ATP and TKIs [45], [48], [49]. According to Yun *et. al.*,[48] who studied the T790M mutation in both active and inactive EGFR structure states, the mutation is hypothesized to alter directly the conformation of the DFG moiety in the ATP-binding pocket from an inactive to active form *via* favourable hydrophobic interactions between M766 and L777, that could lead to changes in the positions of the DFG loop or C-helix.

In terms of comparative binding data, the ATP-binding affinity is higher for the T790M/L858R double mutant than the L858R single mutant. The difference correlates directly with higher resistance towards gefitinib and erlotinib [50]. Here, we reported nearly 32 structures of the T790M/L858R double mutant (with ∼ 23 belonging to the C-helix-IN clan with C-helix-IN (DFGin/BLAminus) conformation, ∼5 structures belonging to C-helix-IN clan with C-helix-IN (DFGin/BLAplus) conformation), and 9 structures of the L858R single mutant found in the C-helix-IN clan with the C-helix-IN/DFGin/BLAminus conformation **(**Supplementary Table 1**)**. Accordingly, we would suggest that the L858R mutation could act to trigger the relay of a “flow of information” from rotamer to rotamer to sensitize EGFR kinase to TKIs. Indeed, this L858R single mutant is a single missense mutation in exon 21 that is also one of the most frequent EGFR alterations found in NSCLC tumours [51]. Moreover, L858R is very frequently mutated to T790M/L858R double mutants in cancer patients, such that other L858R double mutants are found with at best only 5% incidence [52], [53], [54]. Furthermore, if the T790M single mutant and the T790M/L858R double mutant are compared, although they maintain the same low nanomolar affinity for gefitinib as the L858R single mutant, the T790M single mutant exhibits a higher ATP-binding affinity than the L858R single mutant. Accordingly, the T790M/L858R double mutant represents an activated enzyme that becomes resistant to ATP-competitive TKIs [48].

Moving on to the C797S mutation, this is also located in the ATP binding pocket and prevents covalent binding of covalent TKIs. When cysteine is substituted with serine at codon 797, cross-resistance is gained with respect to irreversible third generation TKIs, such as Osimertinib. In this instance, 2 structures, double mutant T790M/C797S and triple mutant T790M/C797S/V948R, appear in the C-helix-OUT clan. The former double mutant from PDB ID 5xgn presents a DFGin/BLAminus conformation with the C-helix axis at equal angles (∼13°) to both 3gop and 1m14 reference structures **(**Supplementary Table 1**)**. The latter triple mutant from PDB ID 5zwj is firmly with C-helix-OUT (DFGin/BLBplus) conformation **(**Supplementary Table 1**).** Accordingly, the C797S mutation could act to trigger the relay of a “flow of information” from rotamer to rotamer to desensitize EGFR kinase to TKIs. In this respect it is worth noting about T790M/C797S double mutations that there are in fact three well described resistance states: 1) the *cis* T790M/C797S allelic state, where both mutations occur in the same receptor protein, which is resistant to all available EGFR-TKIs although sensitive to fourth generation, 2) the *trans* T790M/C797S allelic state, where either of the two expressed receptor proteins harbours one or both mutations, which is sensitive to first and third generation TKIs, 3) a T790M mutation loss combined with a C797S mutation gain which is sensitive to first and the second generation TKIs. Even though the *cis* state dominates, further investigation is needed to understand the structural differences in the ATP binding pocket that are associated with the different mutational combinations [55], [56], [57], [58], [59].

The alternative G719S mutation occurs in the phosphate-binding loop (P-loop) which is considered TKI-sensitive according to the National Comprehensive Cancer Network (NCCN, www.nccn.org) guidelines. Here, 5 cases of the mutant were located in the C-helix-IN clan with active C-helix-IN (DFGin/BLAminus) conformation. In addition, a double mutant G719S/T790M is also located in the C-helix-IN **(**Supplementary Table 1. Arguably, the G719S mutation could act to trigger the relay of a “flow of information” from rotamer to rotamer to sensitize EGFR kinase to TKIs, in this instance. Computational studies on the G719S mutation suggest that TKIs can enter the ATP-binding site with ease. Indeed, simulations indicate that the distance between residues L718 and G796 is increased widening the ATP-binding site for TKIs to enter (conversely, the T790M mutation causes the distance between L718 and G796 to decrease) [60]. Moreover, the G719S mutation destabilizes the inactive conformation and promotes the active conformation of the kinase, leading to more TKI sensitivity [61], [62], [63], [64], [65]. However, when G719S is combined with T790M as a double mutation, the secondary T790M mutation overturns the impact of G719S on the distance between the P-loop and activation loop [60].

Finally, turning to the T790M/C797S/L858R triple mutation, studies on targeted therapy – *via* the new allosteric inhibitor EAI045 in combination with cetuximab – demonstrate a different mode of resistance as compared to that exhibited previously [66], [67]. EAI045 binds allosterically to T790M via a pocket that is facilitated by external dislocation of the C-helix. EAI045 is able to achieve allosteric binding to EGFR by binding to the mutant M790 gatekeeper residue and forming a hydrogen bond with the DFG motif. At least two mechanisms account for the mutant-specificity of the EGFR allosteric inhibitors: Firstly, the M790 gatekeeper residue enhances the selectivity of EAI045 for the T790M mutant. Secondly, in the wildtype EGFR, EAI045 is unable to bind efficiently given the lack of allosteric pocket in the kinase. Cetuximab – a dimerization blocking agent - is usually used with EAI045 to mimic the effect of mutations that disrupt the asymmetric dimer in EGFR. Basically, the allosteric pocket in the L858R/T790M mutant is accessible in the two subunits of the asymmetric dimer unlike in wild type EGFR. Therefore, it is very rational to use cetuximab to enhance the potency of allosteric agents [67].

Our knowledge of another triple EGFR mutant T790M/C797S/V948R comes mainly from comparative binding studies between EAI001 and EAI045. EAI045 exhibits a higher affinity for triple mutants than its predecessor EAI001 for T790M/V948R double mutants [68]. This increased affinity was attributed to the formation of new hydrogen bonds between EAI045 and the backbone of F856. In this case, the C-helix is pushed outwards to accommodate EAI045 binding and the formation of multiple hydrophobic interactions via its aromatic rings (particularly with L747, I759, M766, L777, L788, M790, and F856). On the other hand, further studies are required to shed light on the role of T790M/L858R/V948R triple mutants in EGFR resistance to TKIs. In this instance, it is important to emphasize the role of dimerization dependency in understanding the structure–function consequences of mutations. For example, while several mutants like L858R or G719S are dimerization-dependent (requiring dimerization for oncogenic activation of EGFR), other mutations were reported to be dimerization-independent. Indeed, the V948R mutant represents a surface mutation in the C-lobe and is known as a dimerization-deficient mutant, which is very useful in functional studies [69], [70]. Here we have identified 12 structures belonging to the C-helix-OUT clan with C-helix-OUT (DFGout/BBAminus) conformation **(**Supplementary Table 1**)**. Moreover, all the V948R mutants (single, double and triple) belonged to the C-helix-OUT clan, thus emphasizing its role in inactivation of EGFR kinase. Accordingly, we would suggest that the V948R mutation could act to trigger the relay of a “flow of information” from rotamer to rotamer to desensitize EGFR kinase to TKIs.

### 3.3 Biological significance of EGFR relay system

**Fig. 5:**
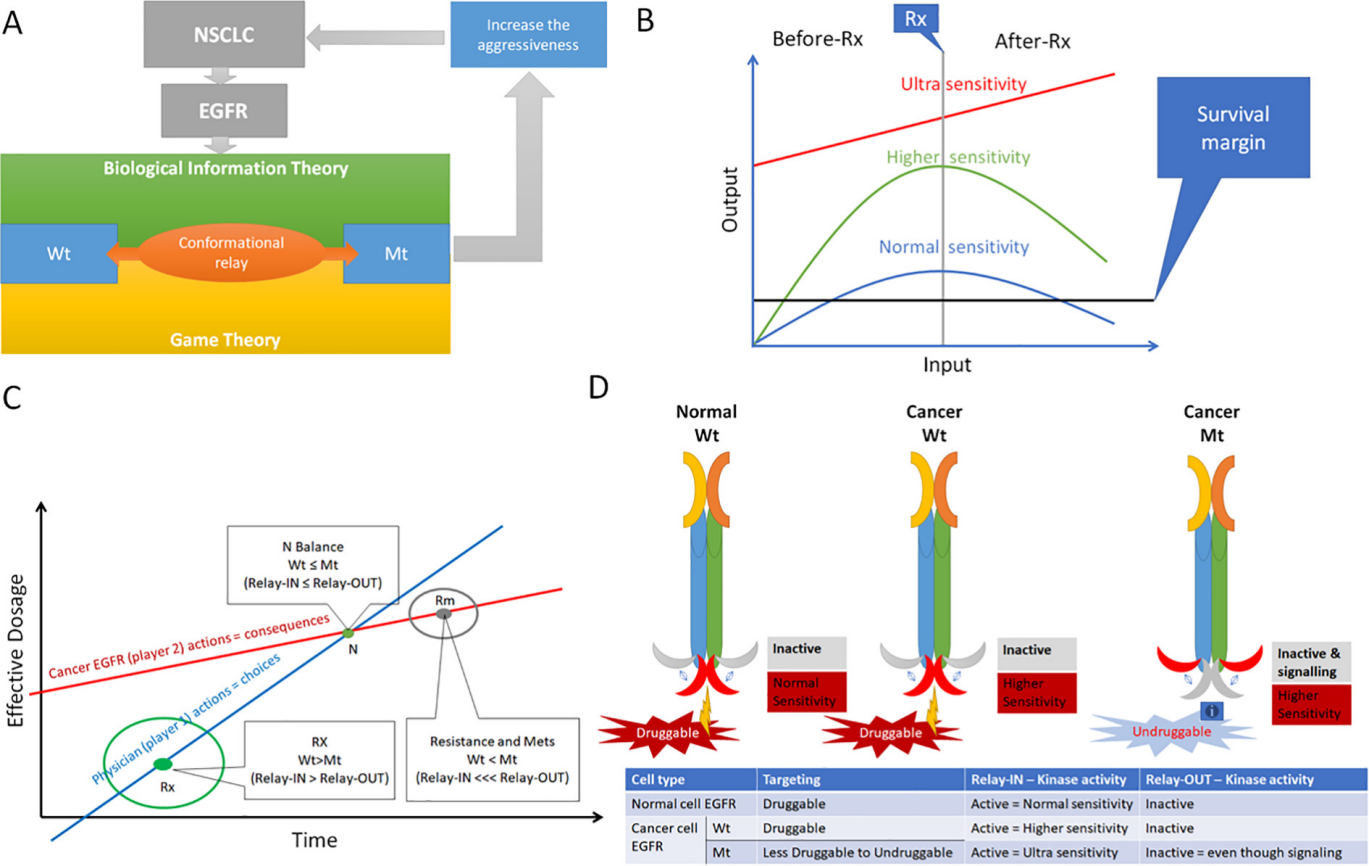
**(A)** A diagram describing our proposed understanding the role of EGFRs (Wt–wildtype and Mt–Mutant) in non-small cell lung carcinoma (NSCLC) tumour resistance to TKIs, with synergistic effects between biological information theory and game theory acting at the molecular level through conformational relays. **(B)** The biological informational theory explanation of conformational relays: this proposed graph describes the different structures of EGFR featuring during different responses to therapy (Rx). Blue = Normal sensitivity = Wt > Mt = (C-helix-IN > C-helix-OUT), Green = Higher sensitivity = Mt ≥ Wt = (C-helix-OUT ≥ C-helix-IN), Red = Ultra sensitivity = Mt >>> Wt = (C-helix-OUT >>> C-Helix-IN). **(C)** A game theory explanation for mutations and conformational relays: the blue line describes the actions of the physicians against the NSCLC, Rx is the point, which describes the most appropriate action of physician as game leader. We propose at this point, that the Wt EGFR mostly in the C-helix-IN conformation exceeds Mt EGFRs. The red line describes the consequences of the physician’s actions on EGFR, N is the Nash balance point where his/her actions start to have no effect on the NSCLC tumour with the formation of C-helix-OUT conformations, Rm is the point where the NSCLC takes the lead from the physician due to his/her repeated actions (therapeutic strategy) that will force the NSCLC to adapt to the new environment. At this point, there will be more mutant C-helix-OUT EGFRs. **(D)** Comparison between different EGFRs in aspects of targeting, druggability, kinase activity/signalling and C-helix position. (For interpretation of the references to color in this figure legend, the reader is referred to the web version of this article.)

EGFR is a part of the signalling processes involved in cell-to-cell communication system [71]. Therefore, this receptor is central to normal as well as cancer cell viability. During chemotherapeutic interventions, EGFR becomes under tremendous selective pressure to maintain its activity, in order to promote cell growth and proliferation. This is clearly demonstrated by the development and subsequent obsolescence of three generations of TKIs through the appearance of a combination of innate (random) and adaptive (induced) EGFR mutations. According to our analysis, EGFR has undergone an adaptive and cumulative sequence of three point mutations which could act singly or together to induce drug resistant conformational changes in EGFR that are communicated by a “flow of information” from rotamer to rotamer *via* side chain conformational relays (Fig. 4, Fig. 5**A**) [72], [73], [74], [75]. Each conformational relay represents a chain of mutation-induced, linked changes (domino-like) in amino acid residue rotamer conformations, that we propose cause the displacement of a whole helix moiety within the tyrosine kinase subdomain (C-helix-OUT). The combined effects of this conformational relay presents a situation where TKI inhibitors no longer have a suitable binding pocket to bind to and inhibit EGFR, and mutant EGFRs themselves become more aggressive agents of signal transduction without the need for typical tyrosine kinase activity [76]. Indeed, such mutant EGFRs preserve the “informational system” with sustained pro-proliferative signalling that is pro cancer cell survival, [38] leading to more aggressive tumour progression than is possible with wildtype EGFR, as observed in NSCLC [77].

The failure of three generations of TKIs to inhibit the EGFR communication system can be understood using Shannon’s Biological Information Theory (Fig. 5**B**) [72], [73], [74], [75]. Briefly, EGFR with a mutant, inactive tyrosine kinase is still capable of transmitting signals and information, hence the kinase function is related to growth not survival [76]. Therefore, in the case of wildtype EGFR drug treatment (Normal sensitivity, Wt > Mt = C-helix-IN > C-helix-OUT), the cell will search for ways to develop drug resistance to maintain growth transmission [36]. Hence, even if the wildtype EGFR has been targeted correctly this will only affect tumour growth temporarily not overall survival [78]. Cancer cells expressing wildtype EGFR do not rely on the EGFR kinase activity but on EGFR for survival [79], [80]. The same is true in the case of the mutant type where EGFR is already undruggable (Higher sensitivity = Mt ≥ Wt = C-helix-OUT ≥ C-helix-IN). In this case, emerging mutations will confer the rotameric relay and further desensitise EGFR to TKIs. Therefore, should physicians insist on using TKIs on EGFR until more mutant inactive EGFR kinases are realized (Ultra sensitivity = Mt >>> Wt = C-helix-OUT >>> C-helix-IN) [81], [82], then the result must be an undruggable protein that will accelerate the signalling cascade, promote cancer cell survival, and worsen disease prognosis [83], [84], [85].

Seen from a different angle, the use of first, second and third generation TKIs leading to the widespread appearance of undruggable mutant EGFRs can be seen and understood through the lens of game theory (Fig. 5**C**) [86], [87]. Cancer cells are known for their adaptivity, but they can neither anticipate nor evolve adaptations for treatments that the physician has not yet applied. Therefore, a distinctive leader–follower (or “Stackelberg”) dynamic must apply, in which the oncologist “leader” plays first and the cancer cell “follower” then responds and adapt to the treatment regime. The physician “plays” a fixed strategy even while the opposing cancer cells continuously evolve counter measures until disease progression is no longer halted [88]. Furthermore, by changing treatment only when the tumour progresses, the physician abandons leadership to the cancer cells and treatment failure becomes nearly inevitable. At the molecular level, we observe the end product of this game theory challenge. In the case of EGFR, obsessive use of one class of drugs for one key target renders the target undruggable as a result of only three mutations and their linked conformational relays (Fig. 5**D**). Our structural and computational data highlight the need to adopt more sophisticated combination approaches for treatment in order to overwhelm tumours before they can mount direct adaptive changes at the molecular level that lead to resistance of treatment. While our structural and computational data account for TKI insensitivity, we do not currently have an equivalent molecular level understanding for how mutant EGFRs possess heightened signal transduction for cancer cell proliferation.

Finally, if we are to look at the conformational relay as equivalent to an electric relay, one should wonder how far the relay “wire” extends? In other words, do rotamer-based conformational relays transfer conformational information beyond the EGFR extracellular domain, or are these relays only for the transmission of relatively local conformational information? Considering that dimerization might play a role in forming the relays via protein–protein interactions, it is unclear if such relays exist in all protein molecules as a mechanism operating via protein–protein interactions. Future work may reveal if such mechanisms are more common or just a unique phenomenon of the kinase domain of EGFR. On the other hand, the literature is full of biological examples where helix movement controls protein functions such as open/close conformations in membrane transporter proteins [89], [90], and enzymes [91]. Furthermore, helix movements are known to facilitate the long-range transmission of conformational change either in case of the closure of clefts between domains in enzymes or in allosteric transitions [92]. We hope that our findings will bring new insights in understanding protein behaviour and also protein folding, particularly taking into consideration that torsional angle movements are the leading switches in protein folding.

## 4 Conclusions

The development of TKIs against the EGFR kinase domain in NSCLC has been a great challenge for researchers in the past two decades. Crystal structures of EGFR kinase domain complexed with ligands have identified several structural conformations, *i.e.*, C-helix-IN/OUT, DFG-in/out, and DFG clusters. Here, we further identified global conformational changes at the side chain level that represent major networks of rotamer-based relays encompassing nearly 43 residues that could correlate with the aforementioned structural conformations. Wildtype apo-EGFR adopts the active C-helix-IN (DFGin/BLAminus) conformation, whereas, wildtype EGFR complexed with ligands adopts both active C-helix-IN (DFGin/BLAminus) and inactive C-helix-OUT (DFGin/BLBplus) conformations. Since single T790M mutants were found in both C-helix-IN and C-helix-OUT clans, and single L858R mutants plus single C719S mutants were found in the C-helix-IN clan, then these mutations appear not to trigger the relay of a “flow of information” from rotamer to rotamer to desensitize EGFR kinase to TKIs. This conclusion is reinforced by the fact that the T790M/L858R double mutant was found mostly in the C-helix-IN clan with C-helix-IN/DFGin conformation (mostly as BLAminus with a few cases reported as BLAplus). Note that we have used the term desensitization as synonymous to inducing C-helix-OUT conformation. In spite of this, the T790M mutation does desensitize EGFR kinase to TKIs through the mechanism of local steric hinderance to drug binding. On the other hand, single C797S and V948R mutants are associated with the C-helix-OUT clan. Accordingly, we suggest that these mutations can trigger the relay of a “flow of information” from rotamer to rotamer to desensitize EGFR kinase to TKIs. Indeed, the T790M/L858R/V948R triple mutant is firmly associated with the C-helix-OUT clan, which underlines the signal importance of the V948R mutation given the variable individual impacts of T790M and L858R mutations, as noted above. The emergence of EGFR mutations that appear to transmit conformational changes by rotamer to rotamer conformational relays to render EGFR undruggable, is an important new concept. Finally, by employing the biological information theory and game theory, we can connect the dots between what is observed in clinic versus what is happening in the cancer microenvironment and its impact on EGFR at the molecular level. Our take-home message for physicians is not to abuse any therapeutic option that might cause loss of lead in the game of cancer therapy. Hence combinatorial approaches to treatment that exploit other weaknesses in the tumour during the early stages of EGFR mutations would be highly appropriate and desirable.

## CRediT authorship contribution statement

**Tareq Hameduh:** Conceptualization, Formal analysis, Funding acquisition, Writing – original draft. **Michal Mokry:** Formal analysis. **Andrew D. Miller:** Conceptualization, Funding acquisition, Project administration, Writing - review & editing. **Vojtech Adam:** Funding acquisition, Project administration. **Zbynek Heger:** Funding acquisition, Project administration, Writing - review & editing. **Yazan Haddad:** Conceptualization, Project administration, Writing - review & editing.

## Declaration of Competing Interest

The authors declare that they have no known competing financial interests or personal relationships that could have appeared to influence the work reported in this paper.
